# Impact of Fatigue on Spine Dynamic Stability and Gait Patterns in Runners with Moderate Flatfoot Versus Normal Arch

**DOI:** 10.3390/bioengineering12111256

**Published:** 2025-11-17

**Authors:** Zihang Xu, Zixiang Gao, Zhanyi Zhou, Yucheng Wang, Jianqi Pan, Liangliang Xiang, Yang Song, Dong Sun, Zsolt Radak, Xuanzhen Cen

**Affiliations:** 1 Faculty of Sports Science, Ningbo University, Ningbo 315211, China; 18503266998@163.com (Z.X.); zhouzhanyi630@hotmail.com (Z.Z.); 2411040024@nbu.edu.cn (Y.W.); 2411040017@nbu.edu.cn (J.P.); sundong@nbu.edu.cn (D.S.); 2 Human Performance Laboratory, Faculty of Kinesiology, University of Calgary, Calgary, AB T2N 1N4, Canada; 3 Department of Physical Education and Sport, Faculty of Sport Sciences, University of Granada, 18071 Granada, Spain; 4 KTH MoveAbility Lab, Department of Engineering Mechanics, KTH Royal Institute of Technology, 114 28 Stockholm, Sweden; liaxi@kth.se; 5 Department of Biomedical Engineering, Faculty of Engineering, The Hong Kong Polytechnic University, Hong Kong 999077, China; yangsong@polyu.edu.hk; 6 Research Institute of Sport Science, Hungarian University of Sport Science, 1123 Budapest, Hungary; radak.zsolt@tf.hu

**Keywords:** flatfoot, running, inertial measurement unit (imu), dynamic stability, fatigue, plantar pressure

## Abstract

**Background:** Running is a widely practiced physical activity but carries a high risk of injury, with foot structure, particularly the medial arch, playing a vital role in biomechanical performance and injury prevention. As the core of foot support, the arch is essential for absorbing impact, transmitting force, and maintaining dynamic stability. This study aims to compare the dynamic stability of runners with moderate flatfoot and those with normal arches in the initial, steady, and fatigue stages in order to elucidate how fatigue differently affects their dynamic postural control. **Methods:** Twelve male runners were recruited. Using inertial measurement units (IMUs) and a Zebris treadmill system, data on Maximum Lyapunov Exponent(MLE) and plantar center of pressure (COP) trajectories were collected during the initial, steady-state, and fatigued phases. **Results:** In the fatigue phase, runners with flatfoot showed an increase of 0.05 s^−1^ in short-term MLE compared to those with normal arches (*p* < 0.05), indicating significantly lower stability under fatigue. **Conclusions:** The deterioration of lower-limb dynamic stability in flatfoot runners is dependent on fatigue. Specifically, their overall lower dynamic stability stems primarily from a marked increase in MLE when entering the fatigued phase. Concurrently, fatigue induces alterations in COP trajectory and temporal gait parameters in flatfoot runners; they signify reduced efficiency in gait control.

## 1. Introduction

As the central axis support structure for human movement, the spine not only bears the body’s own weight but also needs to cope with the periodic loads exerted by the ground reaction force [1]. A stable spine ensures that the torso maintains a reasonable posture during running and coordinates the movement of the upper and lower limbs so as to achieve efficient energy transfer and conversion [2]. In the condition of “spinal muscle fatigue”, the body’s tactile dependence on the foot/ankle increases, resulting in a marked increase in center of pressure sway (COP shift). If flat feet are accompanied by a decrease in the passive stability of the supporting foot (e.g., tension in the Achilles tendon, collapse of the medial arch of the foot, etc.), this shaking will be amplified during spinal fatigue, increasing the load on the lower back muscles, especially the erector spinae and iliocostals, which can easily lead to fatigue, muscle injury, and even chronic injury [3]. A study on runners (including indicators such as the Star Excursion Balance Test and Time-to-Stabilization) found that runners with a history of sports injuries showed significantly increased Vertical Stability Index (VPSI) and Dynamic Postural Stability Index (DPSI) during tasks involving one-legged landing and stabilization. This indicates a decline in their ability to control vertical impact forces, manifested as swaying or delayed adjustments of the torso during landing, which in turn increases the load and injury potential on the lower limb muscle groups [4].

The morphology of the arch of the foot, as a key structure of the foot (e.g., moderately flat feet versus normal arches), significantly affects the biomechanical properties of running [5]. Flat-footed runners tend to have a greater hindfoot valgus angle and velocity in the early stages of running. The study of Barnes et al. clearly measured that pes planus had a significantly higher valgus offset than in the normal or high-arch population, with a simultaneous increase in internal tibial rotation [6]. Flat feet can cause excessive pronation of the foot, which in turn causes internal rotation of the tibia, altering the trajectory and force patterns of the knee and hip joints. This alteration of the lower limb kinematics not only increases the burden on the knee and hip joints but may also affect the stability of the trunk and spine, reducing the efficiency of energy transfer during running. Studies have shown that flat-footed runners are more likely to suffer from knee pain, hip compensation, and sports injuries in the long run [7].

In the state of running fatigue, the flat foot runner has abnormal gait and plantar pressure distribution due to the weakening of the foot support structure, which affects the dynamic stability of the spine. Studies have shown that after fatigue, the pressure in the metatarsal region of the forefoot in low arch runners is significantly increased, and the ground reaction force conduction path is changed, which increases the shear stress of the spine [8]. Another study found that individuals with flat feet increased the trough value of the vertical reaction force after fatigue, decreased the second peak, reduced shock absorption capacity, and more impact force was transmitted to the spine [9]. These changes increase the burden on the core muscles and weaken the dynamic stabilization function of the spine. At the same time, the decrease in spinal dynamic stability can weaken the ability to control the trunk, and in order to maintain balance, runners may unconsciously adjust their gait, resulting in a disordered gait pattern, exacerbating the accumulation of fatigue, and forming a negative feedback loop [10].

Recent studies have confirmed that running fatigue affects the biomechanical properties of the lower extremities and that different arch types (e.g., flat feet vs. normal feet) respond differently to fatigue. For example, Mehrdad et al. found that the plantar forefoot pressure of flatfoot runners increased significantly after fatigue, indicating that the mechanical stability of their lower limbs was more susceptible. Boozari et al. pointed out that the vertical ground reaction force changes more dramatically in individuals with flat feet in the fatigue state, which may increase the burden of body posture control [9]. However, most of these studies focus on lower limb linearity indicators such as plantar pressure and ground reaction force, and there is still a lack of direct assessment of the dynamic stability of the spine, especially the interaction between arch type and fatigue state. In addition, most of the existing literature adopts linear analysis methods, which is difficult to reflect the complexity and nonlinear characteristics of the body control system during running [11]. Studies such as Sanjari, Mehdizadeh et al. have shown that changes in attitude stability can be more sensitively captured using nonlinear dynamical indicators such as the maximum Lyapunov index [11]. Therefore, future studies should combine arch types and fatigue interventions and use nonlinear dynamics methods to explore the mechanism differences in spinal stability in flatfoot runners.

The purpose of this study was to investigate the effect of fatigue on the dynamic stability and gait pattern of the spine in runners with moderately flat feet and normal arches, and to analyze the interaction between arch type and fatigue state in affecting the dynamic stability of the spine. An inertial measurement unit (IMU)-based gait and fatigue analysis method offers significant advantages. Compared to image-based methods (such as biomarker capture and thermal imaging), IMUs enable wearable, real-time, continuous data acquisition. They circumvent issues related to lighting, occlusion, and privacy, making them suitable for natural walking and long-term monitoring environments. Their high temporal resolution and signal stability allow for more precise and reliable quantification of gait spatiotemporal parameters and fatigue-related features, offering high sensitivity and reliability. Therefore, the IMU was primarily used for evaluation in the experiment [12,13,14]. Based on the above analysis, the following hypotheses are proposed: 1. Compared with normal arch runners, moderate flat foot runners have a more significant decrease in spinal dynamic stability under fatigue, and their gait patterns (such as stride length, cadence, landing angle, etc.) are more obvious; 2. There is an interaction between the arch type and the fatigue state in affecting the dynamic stability of the spine; that is, the effect of fatigue on the dynamic stability of the spine varies with different arch types [15]. The results of this study are expected to elucidate the biomechanical and neuromuscular mechanisms underlying the effects of arch type on spinal dynamic stability and gait control under fatigue, thereby providing a theoretical foundation for targeted sports training strategies, injury prevention approaches, and the development of personalized exercise programs for runners with different arch types.

## 2. Materials and Methods

### 2.1. Participants

Twelve male recreational runners (the subjects run ~3–5 km, ~1–2 times per week, on average), consisting of six flatfoot runners and six with normal arches (age: 24.83 ± 1.68 years; height: 171 ± 3.16 cm; weight: 64.92 ± 4.58 kg; foot size: 254.92 ± 9.62 mm; flatfoot arch index: 0.295 ± 0.015). Specifically, calculated from digital footprint analysis obtained via the efoot−350Pros (3DOE Technology Co., Ltd., Shenzhen, China) infrared foot scanner. According to the classification proposed by Cavanagh and Rodgers (1987) and later adopted in multiple biomechanical studies (e.g., Yamashita et al., 2022), an arch index value between 0.26 and 0.32 represents a moderate flatfoot condition [16,17]. Participants had no history of cardiovascular, neurological, or lower limb musculoskeletal disorders, surgeries, or obesity (BMI > 30) [8]. All participants provided informed consent after being informed of the study’s purpose and procedures. Ethical approval was obtained from the Scientific Ethics Committee of Ningbo University (TY2025050).

### 2.2. Experimental Design

Participants were given 10 min to familiarize themselves with the equipment and complete a warm-up. Arch index was assessed using an infrared foot scanner (efoot−350Pros, 3DOE Technology Co., Ltd., Shenzhen, China) before testing. All participants wore identical running shoes [17]. Previous biomechanical studies have shown that different sports shoes substantially alter plantar pressure distribution and COP metrics [18].

**Figure 1:**
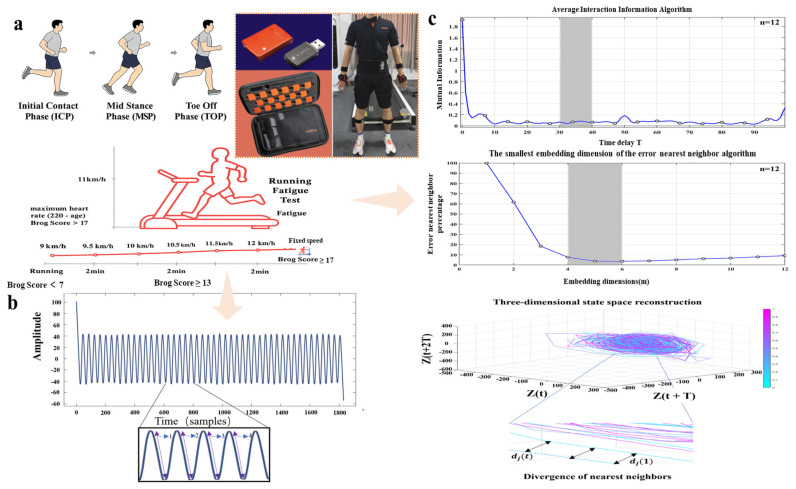
Research workflow. (**a**) We prepare participants to wear the Xsens 17 sensor motion capture suit and Running Fatigue Test. (**b**) shows the results of visualizing Xsens data in Matlab and shows the gait cycle captured using the angular velocity peak detection method. (**c**) Minimum Time delay of the average mutual information algorithm and minimum embedding dimension of the false nearest neighbor’s algorithm, as well as assessment of local dynamic running stability.

Using Xsens MVN Analyze Pro (2024.2) software (Netherlands Xsens Technologies B.V., Enschede, The Netherlands), IMU data were captured in “On-Body Recording Mode” at a sampling rate of 100 Hz. Each sensor captures motion information across three anatomical planes (coronal, sagittal, transverse) and six degrees of freedom [19]. All post-processing of motion measurement data employs the software’s “High-Definition Reprocessing” mode and “No-Level processing scenario”. As shown in Figure 1a, the system comprises 17 sensors integrated into a snug-fitting suit to minimize motion artifacts [20]. Heart rate was monitored with a Polar H10 chest strap and synchronized Polar Beat app [21], placed on the second rib near the heart. Participants ran on a Zebris treadmill (FDM platform, Zebris GmbH, Isni, Germany), sampling frequency set at 100 Hz.

To induce fatigue, The Borg Rating of Perceived Exertion (RPE, 6–20 scale) is a validated and widely applied instrument for quantifying perceived fatigue during physical activity. This scale provides an immediate subjective assessment that demonstrates a strong correlation with key physiological markers such as heart rate and blood lactate concentration, thereby enabling accurate estimation of both physical and muscular fatigue. Compared with visual analogue or multidimensional questionnaires, the Borg 6–20 scale offers superior simplicity, sensitivity, and reproducibility, making it particularly suitable for real-time and field-based monitoring of exertion and fatigue [22]. Therefore, this study assessed fatigue status by using the Borg Scale combined with heart rate monitoring [23]. Running speed was increased by 1 km/h every 2 min until the Borg rating reached level 13 in Figure 1a. Participants then continued running at this pace until Borg level 17 or their theoretical maximum heart rate (220 − age) was achieved [24]. Average running speed was 11.5 ± 2.5 km/h. (The maximum speed is 14 km/h, the minimum speed is 9 km/h, and the average speed is 11.5 km/h.)

### 2.3. Data Collection and Processing

The angular velocity data of gait captured by Xsens undergoes processing through a third-order Butterworth low-pass filter. The filter’s sampling frequency is set to 100 Hz, and its cutoff frequency is set to 20 Hz to achieve zero-phase filtering. This effectively eliminates phase delays, ensuring precise signal processing [25]. Maximum pressure, maximum intensity, contact time ratio, stance phase, swing phase, gait line length, and maximum gait line velocity are collected from runners using the Zebris treadmill (FDM platform, Zebris GmbH, Isni, Germany). These data are then normalized to eliminate the influence of individual differences on stress parameters. Data were acquired during three distinct stages on the treadmill: the initial phase (Gait data on the treadmill during the first 5 min after the start of running), the pre-fatigue steady-state phase (running speed increased incrementally every 2 min until the subject reached their self-perceived maximum acceptable speed), and the post-fatigue phase (Borg index ≥ 17 and maximum heart rate achieved). Continuous 30 s segments of gait data were selected for collection and processing from each of these stages. MLE is calculated based on the spinal angular velocity data acquired by the IMU. The data collected by Xsens were first visualized and subsequently analyzed using MATLAB R2024a. This study employed the MLE as the core metric to quantify Local Dynamic Stability (LDS) during running. LDS reflects an individual’s ability to resist minor internal or external perturbations during continuous motion (i.e., running), meaning the capacity to maintain a predetermined movement pattern and prevent gait instability [26]. A smaller λ value indicates lower sensitivity of the system to minor perturbations and higher dynamic stability; conversely, a larger λ value indicates poorer stability.

Taking the dominant foot (right leg) as an example, angular velocity data from 30 consecutive gait cycles were extracted to assess dynamic stability during running across the specified experimental stages. The gait cycle is defined as the interval between two consecutive heel strikes of the same foot. Also known as stride, it consists of two phases: the stance phase and the swing phase, alternating between legs. The stance phase includes the sequence of contact from heel to toe. The swing phase occurs with the foot suspended in the air [27]. As illustrated in Figure 1b, segmentation of gait cycles was conducted using the angular velocity peak detection method described by Pacini Panebianco et al. [28]. This method, based on a trunk kinematics algorithm, has been shown to provide high accuracy and consistency in identifying gait events and estimating stance duration. All subsequent data processing was performed in MATLAB R2024a. After segmentation, the angular velocity data corresponding to each gait cycle were extracted. To improve the continuity and smoothness of the gait profiles, interpolation was applied. Each of the 30 gait cycles was interpolated to 100 data points, enabling a more temporally refined reconstruction of the original movement patterns.

We used the short-term maximum Lyapunov exponent to evaluate the local dynamic stability of running gait. As illustrated in Figure 1c, MLE quantifies the maximum divergence rate between neighboring trajectories in state space. To specifically highlight differences in dynamic stability induced by fatigue, we divided the running gait into three distinct phases and calculated MLE using the Rosenstein algorithm [29]. A larger MLE value indicates poorer local dynamic stability. For the calculation, we reconstructed the state space from time series data to determine the exponent λ.

(1) $$S\left(t\right)=\left[a_{i}\left(t\right),a_{i}\left(t+\tau \right),a_{i}\left(t+2\tau \right),\ldots ,a_{i}\left(t+\left(m+1\right)\tau \right)\right]$$

Here, “τ” refers to the time delay, “m” represents the embedding dimension, “a” denotes a one-dimensional coordinate, and “i” indicates the sensor axis. The vector S(t) defines the reconstructed m-dimensional state space. The effectiveness of state space reconstruction largely depends on the appropriate selection of “τ” and “m”. In this study, the time delay and embedding dimension for the time series data were determined using the average mutual information algorithm [30] and the global false nearest neighbor algorithm [31], respectively. MLE was then calculated using the Rosenstein algorithm [32], which has been shown to be robust in the presence of experimental noise [33]. Within the reconstructed state space, the algorithm quantifies the Euclidean distance between each point and its initially nearest neighbor according to the following equation.

(2) $$d\left(t\right)=\left\|∆x\left(t\right)\right\|/\left\|∆x\left(0\right)\right\|$$

Thus, the exponential divergence of the *j*-th nearest neighbors from their distance can be expressed as follows.

(3) $$d_{j}\left(i\right)\approx C_{j}*e^{\lambda *(i∆∆)}$$

Here, *C_j_* is a constant representing the initial degree of separation. The *MLE* can therefore be calculated by performing a linear fit to the divergence curve and determining the slope of the line fitted to the logarithm of the average divergence values:

(4) $$MLE=\underset{n\to \infty }{\lim}\;\;\underset{∆x\left(0\right)\to 0}{\lim}\frac{1}{∆t}\left[\frac{∆x\left(t\right)}{∆x\left(0\right)}\right]$$

### 2.4. Statistical Analysis

Statistical analyses were conducted using SPSS version 27.0. Prior to analysis, data were screened for quality: invalid data points, such as missing sensor signals or extreme outliers, were excluded to ensure reliability, and only valid participant data were retained for subsequent analysis. Descriptive statistics are reported as mean ± standard deviation (Mean ± SD). A two-way repeated measures analysis of variance (ANOVA) was performed for the MLE, maximum pressure, maximum force, percentage of contact time, stance phase, swing phase, length of gait line, and maximum gait line velocity. The within-subject factor was the time stage (initial, stable, and fatigued), and the between-subject factor was foot arch type (flatfoot vs. normal arch). The spherical hypothesis was evaluated by the Mauchly test (*p* > 0.05), and if the data violated the Mauchly spherical test, the Greenhouse-Geisser method was used to correct the degrees of freedom [34,35].

## 3. Results

### 3.1. Lyapunov Exponent

Repeated-measures ANOVA showed a significant difference in the main effect on MLE among the initial, stabilization, and fatigue phases, suggesting that there was a significant change in the dynamic stability of the runners in different fatigue phases. The main effect of group (flat feet vs. normal arches) was also significant, with the flat feet group having significantly lower overall dynamic stability than the normal group. In addition, the interaction effect between time phase and group was significant, suggesting that there were differences in the pattern of MLE changes during fatigue between the two groups of runners.

**Figure 2:**
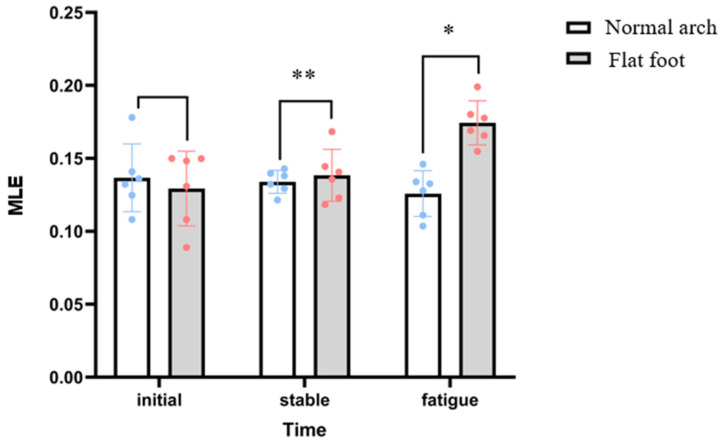
Differences in MLE index between flat feet and normal arches in three stages. Note: (** representative is relatively significant; * representative is significantly prominent.).

The MLE in the flatfoot group was significantly higher in the fatigue phase (0.17 ± 0.02 s^−1^) than in the initial phase (0.13 ± 0.02 s^−1^) and in the stabilization phase (0.14 ± 0.02 s^−1^), whereas there was no significant difference between the phases in the normal group. In the fatigue stage, the MLE value of the flatfoot group was significantly higher than that of the normal group, while the differences in the other stages were not significant, as shown in Figure 2.

### 3.2. Center of Pressure (COP) Parameters

**Figure 3:**
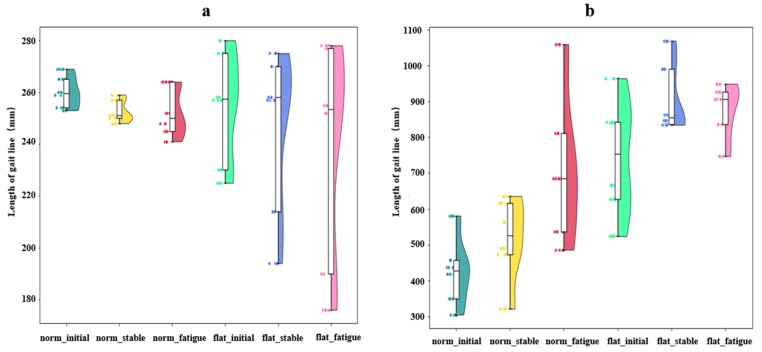
(**a**) Differences in COP trajectory length between flat feet and normal arches in three stages; (**b**) differences in COP trajectory velocity between flat feet and normal arches in three stages.

Repeated-measures ANOVA showed a significant main effect of time phase (initial, stabilization, fatigue) on COP trajectory speed during exercise, as shown in Figure 3a. The main effect of COP trajectory length during exercise was also significant. The main effect of the group (flat feet vs. normal arches) was also significant, as shown in Figure 3b. The overall center of pressure trajectory of the flat feet group had a significantly higher dispersion value than that of the normal group and was more unstable.

### 3.3. Plantar Mechanics Parameters

As shown in Table 1, significant differences were observed in key biomechanical indicators between groups.

In terms of temporal parameters, the stance phase ratio was comparatively shorter in the flatfoot group after fatigue. Midfoot contact time was longer in the flatfoot group, suggesting reliance on midfoot support during fatigue. The ratio of the swing phase has relatively increased, reflecting a decline in gait control efficiency and a change in gait rhythm.

As can be seen in Table 1, moderate flatfoot runners before and after fatigue had greater dispersion values in terms of maximal force, maximal pressure, and percentage of contact time before and after fatigue, showed significant pressure distribution imbalance, increased compensatory force, decreased gait control, and had inferior dynamic stability compared to runners with normal arches.

## 4. Discussion

This study primarily investigates the dynamic stability of runners with mild flat feet and normal arches before and after fatigue. By analyzing the impact of fatigue on the dynamic stability of runners with different foot arch types, it aims to understand the specific differences in how their dynamic stability changes. The results indicate distinct differences between the two arch types. The treadmill-based experimental protocol revealed that only in the flatfoot group did the short-term MLE increase significantly, indicating heightened sensitivity to minor perturbations and impaired local dynamic stability. Furthermore, only flatfoot runners exhibited prolonged COP trajectory after fatigue. Although maximum force decreased after fatigue in both arch types, this likely reflects a general reduction in muscular output due to neuromuscular fatigue [36,37]. In contrast, maximum pressure showed divergent trends between groups because pressure depends on both total force and contact area. Normal-arch runners maintained relatively uniform load distribution after fatigue, whereas flat-footed runners exhibited arch collapse and medial-forefoot load concentration, leading to higher localized peak pressures despite the reduced total force [8,9].

A unique compensatory pattern was also identified in flatfoot runners’ post-fatigue, characterized by increased forefoot pressure, prolonged midfoot contact time, and shortened stance phase, reflecting arch collapse and reduced gait efficiency. Notably, significant differences between the groups were not observed during the initial or steady-state phases but emerged primarily during the fatigue phase. Kinematic data for flatfoot runners also showed substantial changes compared to normal-arch runners during fatigue. Under fatigue, flatfoot runners compensated for diminished proprioception by increasing their COP range of motion, reflected in longer COP trajectory lengths and higher COP velocities.

These plantar pressure mechanic findings align with Song et al. [38], who reported significantly higher post-exercise COP velocity, COP path length, and contact area in flatfoot runners compared to the normal-arch group, consistent with this study’s hypothesis. Unlike many previous studies that relied mainly on linear biomechanical indicators, this study integrates nonlinear dynamic stability metrics with spatiotemporal gait and plantar pressure analyses. This comprehensive approach reveals how fatigue-induced dynamic instability uniquely affects flatfoot runners, offering new insights into the interaction between fatigue and foot morphology.

According to Rosenstein’s theory, the MLE value reflects a system’s sensitivity to small perturbations, and an increase in this value indicates reduced predictability of the gait trajectory [39]. This phenomenon is closely linked to diminished neuromuscular control efficiency in flat-footed runners: collapse of the medial longitudinal arch reduces foot stiffness, necessitating compensatory contractions of the plantar fascia and tibialis posterior muscles to maintain stability [40]. However, fatigue impairs the muscles’ ability for sustained contraction, further weakening arch support and limiting gait adjustment capacity [36]. Furthermore, compensatory effects within the kinetic chain may exacerbate instability. Flat-footed runners compensate for insufficient foot stability by increasing knee flexion or hip internal rotation [41]. While this temporarily maintains athletic performance, the increased movement variability leads to higher MLE values. Under fatigue conditions, flat-footed runners offset diminished proprioception by expanding their COP displacement range, manifesting as longer COP trajectory lengths and elevated COP velocities. This study comprehensively employs MLE, COP trajectory length and COP velocity (center of pressure adjustment amplitude and rate), and plantar COP displacement rate (dynamic equilibrium of pressure distribution) to multidimensionally quantify the impact of fatigue on gait and postural control. This provides quantitative evidence for developing personalized training and intervention programs. It should be noted that the study is limited by its relatively small sample size of 12 male participants, which may restrict the generalizability of the findings to broader populations, including females. In addition, the study did not differentiate between flatfoot subtypes (such as flexible and rigid), which may further limit the specificity of the conclusions. Female biomechanics may differ significantly due to variations in pelvic width and Q-angle [40]. The fatigue protocol, graded treadmill running to Borg 17, did not account for psychological or environmental fatigue factors [42]. Long-term effects, such as sustained compensations potentially increasing the risk of plantar fasciitis, were not examined. Future research should explore dose–response relationships between arch morphology and stability, incorporating imaging (e.g., MRI) and multimodal data (e.g., EMG), and further examine whether customized flat-foot insoles or orthotic interventions can effectively reduce MLE deviations in fatigued conditions by improving medial arch support and redistributing plantar pressure. Longitudinal trials combining nonlinear dynamics and plantar pressure assessment could provide insight into how orthotic correction influences spine–lower limb interaction and fatigue resilience in flatfoot runners. Longitudinal trials are necessary to verify the effectiveness of orthotics or neuromuscular interventions in reducing injury incidence and improving performance.

Fatigue is a key factor in dynamic instability. Real-time monitoring should combine the Borg scale with biomechanical measures such as COP speed; for example, a COP speed above 800 cm/s may indicate critical fatigue. Staged recovery strategies, like taking 1 min walking breaks every 15 min during long-distance running, help reduce cumulative plantar muscle fatigue [36]. To address flat foot structural issues, both support and neuromuscular interventions are necessary. Future research should further investigate whether customized orthotic insoles and specialized running shoes for flat-footed runners can enhance medial arch support and optimize plantar pressure distribution. Such interventions may help alleviate excessive pronation and potentially reduce the MLE deviations observed under fatigue conditions. In addition, systematic reviews have shown that unstable/“bionic” footwear designs can modify COP behavior, muscle activation, and plantar pressure patterns and thus may be used as an adjunct to training or orthotics to improve dynamic stability [43]. Attention should be given to potential inter-limb asymmetries in running kinematics and foot morphology [41,44,45]. Additionally, training programs that include balance exercises on pads, resisted plantar flexion, and reactive jumping can strengthen the posterior tibialis muscle and plantar fascia [37,46]. For instance, visual interference training, such as tracking a moving target during single-leg stance, enhances proprioception and dynamic control simultaneously.

## 5. Conclusions

This study systematically revealed the fatigue-induced decline in dynamic stability in runners with moderate flatfoot using MLE, COP metrics, and plantar biomechanics. Fatigue may impair neuromuscular control, induce compensatory kinematics, and disrupt load balance—significantly reducing gait stability in flatfoot runners. It is recommended to adopt multi-dimensional quantitative monitoring during training and rehabilitation, such as assessing local dynamic stability, COP characteristics, plantar pressure distribution, and gait parameters, and to develop individualized intervention programs accordingly, including balance/proprioception training, muscle strengthening, orthotic support, or gait modification. Regular re-evaluation should be conducted to dynamically adjust interventions in order to enhance therapeutic effectiveness and reduce the risk of sports-related injuries.

## Figures and Tables

**Table 1:** Plantar mechanical parameters before fatigue and after fatigue in flat feet and normal arches.

Indicator	Flat Feet Group (Forefatigue)	Flat Feet (Afterfatigue)	Normal Group (Forefatigue)	Normal Group (Afterfatigue)	F	*p*
*MLE* (s^−1^)	0.129 ± 0.01	0.174 ± 0.006	0.134 ± 0.008	0.126 ± 0.006	8.365	0.020
Maximum pressure (N/cm^2^)	16.5 ± 2.05	16.9 ± 2.2	12.4 ± 0.9	11.8 ± 0.9	14.848	0.003
Maximum force (N)	400 ± 29.8	394.7 ± 47.8	396.5 ± 31.2	384.5 ± 29.2	8.881	0.014
Contact time (%) of stance time (Midfoot)	61.9 ± 3.1	63.5 ± 2.9	60.2 ± 2.2	61.4 ± 2.8	6.120	0.033
Stance phase (%)	38.75 ± 4.99	37.12 ± 4.08	37.92 ± 3.41	34.5 ± 1.03	4.92	0.048
Swing phase (%)	61.23 ± 4.97	62.88 ± 4.08	62.08 ± 3.43	65.5 ± 1.03	5.03	0.046
Length of gait line (mm)	244.6 ± 12.9	230.6 ± 13.5	259.9 ± 5.6	252.3 ± 7.8	4.996	0.049
Max gait line velocity (cm/sec)	744.6 ± 162.5	932.3 ± 161.2	424.6 ± 95.7	710.7 ± 207.1	18.723	0.001

Note: MLE (Maximum Lyapunov Exponent).

## Data Availability

The raw data supporting the conclusions of this article will be made available by the authors on request.

## Abbreviations

The following abbreviations are used in this manuscript:

MLE	Maximum Lyapunov Exponent
IMUs	Inertial measurement units
COP	Center of pressure
VPSI	Vertical Postural Stability Index
DPSI	Dynamic Postural Stability Index
LDS	Local Dynamic Stability

## References

[1] Granata K.P., England S.A. (2006). Stability of dynamic trunk movement. Spine.

[2] Teng H.L., Powers C.M. (2015). Influence of trunk posture on lower extremity energetics during running. Med. Sci. Sports Exerc..

[3] Vuillerme N., Pinsault N. (2007). Re-weighting of somatosensory inputs from the foot and the ankle for controlling posture during quiet standing following trunk extensor muscles fatigue. Exp. Brain Res..

[4] Meardon S., Klusendorf A., Kernozek T. (2016). Influence of injury on dynamic postural control in runners. Int. J. Sports Phys. Ther..

[5] Nachbauer W., Nigg B.M. (1992). Effects of arch height of the foot on ground reaction forces in running. Med. Sci. Sports Exerc..

[6] Williams D.S., McClay I.S., Hamill J., Buchanan T.S. (2001). Lower Extremity Kinematic and Kinetic Differences in Runners with High and Low Arches. J. Appl. Biomech..

[7] Ataabadi P.A., Abbassi A., Letafatkar A., Vanwanseele B. (2022). The effects of foot orthosis and low-dye tape on lower limb joint angles and moments during running in individuals with pes planus. Gait Posture.

[8] Anbarian M., Esmaeili H. (2016). Effects of running-induced fatigue on plantar pressure distribution in novice runners with different foot types. Gait Posture.

[9] Boozari S., Jamshidi A.A., Sanjari M.A., Jafari H. (2013). Effect of Functional Fatigue on Vertical Ground-Reaction Force in Individuals With Flat Feet. J. Sport Rehabil..

[10] Glover N.A., Chaudhari A.M.W. (2024). Neuromuscular and trunk control mediate factors associated with injury in fatigued runners. J. Biomech..

[11] Mehdizadeh S., Sanjari M.A. (2017). Effect of noise and filtering on largest Lyapunov exponent of time series associated with human walking. J. Biomech..

[12] Lee Y.J., Wei M.Y., Chen Y.J. (2022). Multiple inertial measurement unit combination and location for recognizing general, fatigue, and simulated-fatigue gait. Gait Posture.

[13] Boutaayamou M., Pelzer D., Schwartz C., Gillain S., Garraux G., Croisier J.L., Verly J.G., Brüls O. (2025). Toward Convenient and Accurate IMU-Based Gait Analysis. Sensors.

[14] Prasanth H., Caban M., Keller U., Courtine G., Ijspeert A., Vallery H., von Zitzewitz J. (2021). Wearable Sensor-Based Real-Time Gait Detection: A Systematic Review. Sensors.

[15] Larson D.J., Pinto B.L., Brown S.H.M. (2018). Differential effects of muscle fatigue on dynamic spine stability: Implications for injury risk. J. Electromyogr. Kinesiol..

[16] Cavanagh P.R., Rodgers M.M. (1987). The arch index: A useful measure from footprints. J. Biomech..

[17] Yamashita T., Yamashita K., Sato M., Kawasumi M., Ata S. (2022). Analysis of skeletal characteristics of flat feet using three-dimensional foot scanner and digital footprint. Biomed. Eng. Online.

[18] Mei Q., Graham M., Gu Y. (2014). Biomechanical analysis of the plantar and upper pressure with different sports shoes. Int. J. Biomed. Eng. Technol..

[19] Qian Y., Sun D., Xia Z., Shao E., Song Y., Sárosi J., Bíró I., Gao Z., Gu Y. (2025). Estimating dynamic plantar pressure distribution from wearable inertial sensors using a hybrid CNN-BiLSTM architecture. Acta Bioeng. Biomech..

[20] Di Paolo S., Santillozzi F., Zinno R., Barone G., Bragonzoni L. (2022). On-Field Biomechanical Assessment of High and Low Dive in Competitive 16-Year-Old Goalkeepers through Wearable Sensors and Principal Component Analysis. Sensors.

[21] Walecka I., Gąsior J.S., Wieniawski P., Werner B. (2024). Elite HRV smartphone application using Polar H10 is valid for short-term heart rate variability analysis in pediatric cardiac patients. Kardiol. Pol..

[22] Scherr J., Wolfarth B., Christle J.W., Pressler A., Wagenpfeil S., Halle M. (2013). Associations between Borg’s rating of perceived exertion and physiological measures of exercise intensity. Eur. J. Appl. Physiol..

[23] Gimunová M., Bozděch M., Bernaciková M., Fernandes R., Kumstát M., Paludo A. (2024). The relationship between low energy availability, injuries, and bone health in recreational female athletes. PeerJ..

[24] Gao Z., Fekete G., Baker J.S., Liang M., Xuan R., Gu Y. (2022). Effects of running fatigue on lower extremity symmetry among amateur runners: From a biomechanical perspective. Front. Physiol..

[25] Bötzel K., Marti F.M., Rodríguez M., Plate A., Vicente A.O. (2016). Gait recording with inertial sensors--How to determine initial and terminal contact. J. Biomech..

[26] Kennerley A.S., Dunn M., Middleton K., Webster K.E., Wheat J. (2025). The effect of run duration, gait variable and Lyapunov exponent algorithm on the inter-session reliability of local dynamic stability in healthy young people. Hum. Mov. Sci..

[27] Gao S., Song Y., Sun D., Cen X., Wang M., Lu Z., Gu Y. (2025). Impact of Becker muscular dystrophy on gait patterns: Insights from biomechanical analysis. Gait Posture.

[28] Pacini Panebianco G., Bisi M.C., Stagni R., Fantozzi S. (2018). Analysis of the performance of 17 algorithms from a systematic review: Influence of sensor position, analysed variable and computational approach in gait timing estimation from IMU measurements. Gait Posture.

[29] Xiang L., Gu Y., Wang A., Mei Q., Yu P., Shim V., Fernandez J. (2022). Effect of foot pronation during distance running on the lower limb impact acceleration and dynamic stability. Acta Bioeng. Biomech..

[30] Fraser A.M., Swinney H.L. (1986). Independent coordinates for strange attractors from mutual information. Phys. Rev. A Gen. Phys..

[31] Kennel M.B., Brown R., Abarbanel H.D. (1992). Determining embedding dimension for phase-space reconstruction using a geometrical construction. Phys. Rev. A..

[32] Raffalt P.C., Kent J.A., Wurdeman S.R., Stergiou N. (2019). Selection Procedures for the Largest Lyapunov Exponent in Gait Biomechanics. Ann. Biomed. Eng..

[33] Mehdizadeh S. (2018). The largest Lyapunov exponent of gait in young and elderly individuals: A systematic review. Gait Posture.

[34] Mauchly J.W. (1940). Significance Test for Sphericity of a Normal n-Variate Distribution. Ann. Math. Stat..

[35] Abdi H. (2010). The greenhouse-geisser correction. Encycl. Res. Des..

[36] Paillard T. (2012). Effects of general and local fatigue on postural control: A review. Neurosci. Biobehav. Rev..

[37] Bercovitz T., Herman A., Solomonow-Avnon D., Wolf A., Kodesh E. (2022). Plantar pressure modifications in experienced runners following an exhaustive run. Sports Biomech..

[38] Song J.-Y., Park S.-H., Lee M.-M. (2021). The Comparison of the Difference in Foot Pressure, Ground Reaction Force, and Balance Ability According to the Foot Arch Height in Young Adults. Original Article. Ann. Appl. Sport Sci..

[39] Rosenstein M.T., Collins J.J., De Luca C.J. (1993). A practical method for calculating largest Lyapunov exponents from small data sets. Phys. D Nonlinear Phenom..

[40] Buldt A.K., Murley G.S., Butterworth P., Levinger P., Menz H.B., Landorf K.B. (2013). The relationship between foot posture and lower limb kinematics during walking: A systematic review. Gait Posture.

[41] Chang J.S., Kwon Y.H., Kim C.S., Ahn S.H., Park S.H. (2012). Differences of ground reaction forces and kinematics of lower extremity according to landing height between flat and normal feet. J. Back. Musculoskelet. Rehabil..

[42] Borg G.A. (1982). Psychophysical bases of perceived exertion. Med. Sci. Sports Exerc..

[43] Zhou H., Ugbolue U.C. (2024). Biomechanical Analysis of Lower Limbs Based on Unstable Condition Sports Footwear: A Systematic Review. Phys. Act. Health.

[44] Pan J.W., Ho M.Y.M., Loh R., Iskandar M.N.S. (2023). Foot morphology and running gait pattern between the left and right limbs in recreational runners. Phys. Act. Health.

[45] Song Y., Cen X., Wang M., Gao Z., Tan Q., Sun D., Gu Y., Wang Y., Zhang M. (2025). A systematic review of finite element analysis in running footwear biomechanics: Insights for running-related musculoskeletal injuries. J. Sports Sci. Med..

[46] Gribble P.A., Hertel J., Plisky P. (2012). Using the Star Excursion Balance Test to assess dynamic postural-control deficits and outcomes in lower extremity injury: A literature and systematic review. J. Athl. Train..

